# Familial history and prevalence of *BRCA1*, *BRCA2* and *TP53* pathogenic variants in HBOC Brazilian patients from a public healthcare service

**DOI:** 10.1038/s41598-022-23012-3

**Published:** 2022-11-03

**Authors:** Bruna Palma Matta, Renan Gomes, Daniel Mattos, Renata Olicio, Caroline Macedo Nascimento, Gerson Moura Ferreira, Ayslan Castro Brant, Mariana Boroni, Carolina Furtado, Valdirene Lima, Miguel Ângelo Martins Moreira, Anna Cláudia Evangelista dos Santos

**Affiliations:** 1 Programa de Genética e Virologia Tumoral, Instituto Nacional de Câncer (INCA), Rio de Janeiro, Brazil; 2 Programa de Pós-Graduação em Genética, Universidade Federal do Rio de Janeiro (UFRJ), Rio de Janeiro, Brazil; 3 Centro de Transplante de Medula Óssea, Instituto Nacional de Câncer (INCA), Rio de Janeiro, Brazil; 4 Laboratório de Bioinformática e Biologia Computacional, Instituto Nacional de Câncer (INCA), Rio de Janeiro, Brazil; 5 Present Address: Hereditary Cancer Genetics Group, Vall d’Hebron Institute of Oncology (VHIO), Vall d’Hebron Barcelona Hospital Campus, Barcelona, Spain; 6 Present Address: Tumor Virus RNA Biology Section, HIV DRP, National Cancer Institute, NIH, Frederick, MD USA

**Keywords:** Cancer, Genetics, Molecular biology, Diseases, Health care, Medical research, Molecular medicine, Oncology, Risk factors

## Abstract

Several studies have demonstrated the cost-effectiveness of genetic testing for surveillance and treatment of carriers of germline pathogenic variants associated with hereditary breast/ovarian cancer syndrome (HBOC). In Brazil, seventy percent of the population is assisted by the public Unified Health System (SUS), where genetic testing is still unavailable. And few studies were performed regarding the prevalence of HBOC pathogenic variants in this context. Here, we estimated the prevalence of germline pathogenic variants in *BRCA1*, *BRCA2* and *TP53* genes in Brazilian patients suspected of HBOC and referred to public healthcare service. Predictive power of risk prediction models for detecting mutation carriers was also evaluated. We found that 41 out of 257 tested patients (15.9%) were carriers of pathogenic variants in the analyzed genes. Most frequent pathogenic variant was the founder Brazilian mutation *TP53* c.1010G > A (p.Arg337His), adding to the accumulated evidence that supports inclusion of *TP53* in routine testing of Brazilian HBOC patients. Surprisingly, *BRCA1* c.5266dupC (p.Gln1756fs), a frequently reported pathogenic variant in Brazilian HBOC patients, was not observed. Regarding the use of predictive models, we found that familial history of cancer might be used to improve selection or prioritization of patients for genetic testing, especially in a context of limited resources.

## Introduction

Hereditary breast and ovarian cancer (HBOC) is an inherited cancer-predisposition syndrome in which individuals have increased risk of developing breast cancer (BC), including male BC, ovarian cancer (OC), pancreatic cancer, prostate cancer, and other malignancies. Even though the majority of BC and OC cases are sporadic, approximately 5–10% are HBOC cases associated with germline pathogenic or likely pathogenic variants (PV or LPV, respectively) in genes related to the homologous recombination DNA repair and tumor suppressor genes, foremost *BRCA1* and *BRCA2*^1–6^. Up to the age of 80, women harboring PV/LPV in *BRCA1* and *BRCA2* genes have a cumulative risk of 72% and 69% for developing BC, and of 44% and 17% for developing OC, respectively, compared to 12% lifetime risk of developing BC and 1% of developing OC for women in the general population^7^. Breast cancer is also the most frequently observed cancer in women carrying germline variants in *TP53* gene, another high-penetrance tumor suppressor gene, generally associated with early-onset cases (age of diagnosis ≤ 40 years) and HER2 positive breast tumors^8–11^*.* In Brazilian patients presenting hereditary BC, the prevalence of *TP53* PV/LPV may reach up to 8.6%, depending on the clinical criteria adopted for patient selection^12–17^.

Over the past years, deleterious variants associated with hereditary BC in the Brazilian population have been increasingly reported, especially due to the decreasing costs of DNA sequencing and adoption of massive parallel sequencing technologies^12,15^. Thus far, *BRCA1/2* variants observed in Brazilian populations are shown to be highly heterogeneous^15,18,19^, substantially differing from *BRCA1/2* variants in other populations^20^. On the other hand, *TP53* variants are shown to be less heterogeneous^12,15,21^, and one particular variant was found to be relevant in Brazil: the founder mutation p.Arg337His, a variant detected in 0.3% of the inhabitants of South and Southeast Brazilian regions^12,13,15,16,21^.

Nevertheless, the knowledge on PV/LPV in hereditary BC and OC cases in Brazilian populations remains limited, especially due to limitations of the Brazilian healthcare system. Several studies have demonstrated the cost-effectiveness of genetic testing, regarding surveillance and treatment strategies of *BRCA1/2* PV/LPV carriers^22–24^. However, genetic testing is still unavailable for high-risk patients in the context of Brazilian public Unified Health System (SUS), which provides medical assistance to approximately 70% of the population, particularly to low- and middle-income patients^25–27^. Contrariwise, access to genetic testing in the Brazilian private healthcare system is regulated by the National Agency of Supplementary Health (ANS). Following ANS regulatory guidelines, private healthcare companies began to provide *BRCA1/2* testing for patients with personal or familial history suggestive of HBOC in 2014^28^, and for other HBOC associated genes in 2018^29^. The recent and limited access to genetic screening for cancer risk assessment in Brazil reflects in under-reporting of individuals diagnosed with hereditary cancer^12^ and their associated genetic variants.

Historically, the high costs associated with genetic testing have led to the establishment of selection criteria in order to identify patients suspected of HBOC with higher chances of harboring PV/LPV. Criteria such as the Clinical Practice Guidelines of National Comprehensive Cancer Network (NCCN)^30^, as well as risk prediction models^31^ have been used to identify patients and families at risk for HBOC, which could fully benefit from genetic testing and genetic counseling. However, there is no appropriate threshold derived from these predictive models that could be used in the selection of high-risk patients.

Taking into account that the knowledge on the spectrum of genetic variants associated with HBOC may help to determine more appropriate screening and patient management strategies, the purposes of this study were: to estimate the prevalence of PV/LPV in *BRCA1*, *BRCA2* and *TP53* genes in Brazilian HBOC patients referred to a public healthcare service for cancer treatment; to evaluate the associations of PV/LPV with clinical features; to evaluate possible benefits of including *TP53* in routine genetic testing; and to evaluate the predictive power of risk prediction models and familial history of cancer in this cohort. Our main findings indicate that including *TP53* in routine genetic testing of HBOC Brazilian patients might be worthwhile, and that familial history of cancer might outperform risk prediction models in predicting PV/LPV carriers, at least for *BRCA2*.

## Results

### Clinicopathological data of index patients

**Figure 1:**
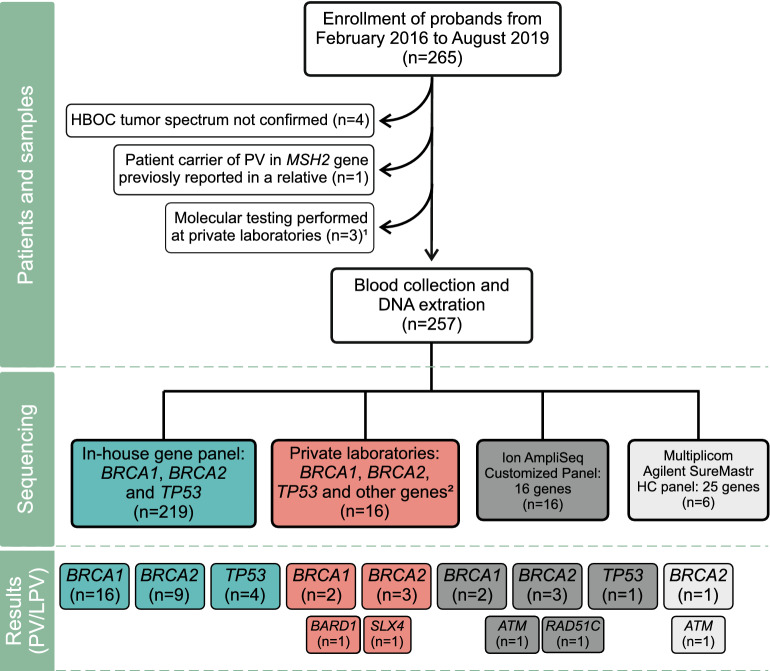
Study design and main results. This flowchart depicts the criteria for patient inclusion/exclusion of patients, the distribution of samples across the different massive parallel sequencing methodologies, as well as the results of pathogenic and likely pathogenic variants (PV/LPV) detected in this study. ^1^Results not provided. ^2^Private laboratories: eight patients were sequenced using gene panels that included *BRCA1/2* and *TP53* (panels with 16 to 207 genes); and eight patients were sequenced only for *BRCA1/2* genes, while *TP53* was later sequenced through Sanger sequencing at our laboratory, except for one patient for which DNA sample was not available. In total, 227 patients were sequenced only for *BRCA1/2* and *TP53* genes (219 in-house and eight at private laboratories), while only 30 patients were sequenced for genes beyond *BRCA1/2* and *TP53*.

This study consists of a consecutive cohort of patients suspected of HBOC that were referred to the Genetic Counseling Program of the Brazilian National Cancer Institute (INCA) from February 2016 to August 2019 and were enrolled if at least one NCCN criterion^32^ was fulfilled (Fig. 1). A total of 257 patients were included in the cohort, 248 women and 9 men, and clinical data are summarized in Table 1. The average age at diagnosis of the first tumor was 41.1 years (range 13–88 years), and the average age at diagnosis of the first breast, ovarian, pancreatic, or prostate cancer (hereafter named HBOC core tumors, for convenience) was 41.6 years (range 13–88 years). Only 16 patients (all women) presented first tumors other than HBOC core tumors (Supplementary Table S1). The nine men in the cohort had BC (eight cases) or pancreatic cancer (one case) as first malignancy, and none presented personal history of prostate cancer.

**Table 1:** Study cohort; clinical and familial data according to mutational status of *BRCA1/2* and *TP53* genes.

*Clinical data*	Positive (n = 41)	%	Negative (n = 216)	%	*p*
**Sex**					0.159
Female	38	93	210	97	
Male	3	7	6	3	
**First HBOC core cancer**					0.515
Breast	39	95	209	97	
Ovarian	2	5	5	2	
Pancreatic	0	0	2	1	
**Multiple tumors**					0.102
Yes	13	32	43	20	
No	28	68	173	80	
**Age at first HBOC core cancer**					0.547
< 30	7	17	24	11	
30–39	16	39	87	40	
40–49	9	22	50	23	
≥ 50	9	22	55	25	
Mean (SD)	40.6 (11.3)		41.8 (12.3)		
Median	39		39		

Familial data of FH(+) patients*	Positive (n = 30)		Negative (n = 137)		*p*
**Relatives with cancer** Mean (SD)	3.6 (1.7)		2.8 (1.8)		**0.024**
**Relatives with HBOC core cancer** Mean (SD)	2.6 (1.4)		2.0 (1.3)		0.057
**Relatives with other cancer** Mean (SD)	1.0 (1.2)		0.8 (1.3)		0.232
**Family size** Mean (SD)	25.4 (16.8)		22.7 (12.1)		**0.004**

Positive/negative refers to the presence/absence of a pathogenic or likely pathogenic variant (PV/LPV) in *BRCA1*, *BRCA2* or *TP53* genes. SD = standard deviation. For convenience, HBOC core cancer refers to breast, ovarian, pancreatic, and prostate cancer; but no prostate cancer was observed in the index patients. * Familial data of patients with FH(+) excludes 90 patients (FH(−)) that did not fulfill NCCN criteria for familial history of HBOC. Fisher’s exact test was applied to all variables, except for analyses of age (where T-test was applied), and familial data (where Poisson regression was applied). All *p*-values < 0.05 are in bold.

A total of 248 BC cases were observed in the probands, and the majority of cases were diagnosed as first tumor (232/257 = 90.3%) or as first HBOC core tumor (14/257 = 5.4%), while two BC cases were diagnosed as second or fourth tumor. Clinical data of BC cases are summarized in Table 2. Most BC patients presented unilateral tumors (216/248 = 87.1%). The predominant type was invasive ductal carcinoma (IDC) (225/248 = 90.7%). Regarding receptor status, 179 were ER/PR + (72.2%), 14 were HER2 + (5.6%), and 44 were triple negative (17.7%). One hundred and eighty-four BC cases (74.2%) presented increased Ki-67 expression. At the time of BC diagnosis, 96 patients had localized cancer (38.7%; stages 0, I, or IIA), 140 had regional cancer (56.5%; stages IIB or III) and 7 had metastatic cancer (2.8%; stage IV). Grade 2 was the predominant Nottingham histological score for BC cases (136/248 = 54.8%).

**Table 2:** Breast cancer patients; clinical data according to mutational status of *BRCA1/2* and *TP53* genes.

Clinical data	Positive (n = 39)	%	Negative (n = 209)	%	*p*
**Sex**					0.114
Female	36	92	204	98	
Male	3	8	5	2	
**Age at first BC**					0.523
< 30	7	18	21	10	
30–39	15	38	85	41	
40–49	9	23	49	23	
≥ 50	8	21	54	26	
Mean (SD)	39.9 (10.9)		42.1 (12.1)		
Median	38		39		
**Laterality**					**0.007**
Unilateral	28	72	188	90	
Bilateral	11	28	21	10	
**Stage**					0.225
Localized	13	33	83	40	
Regional	22	56	118	56	
Distant	2	5	5	2	
na	2	5	3	1	
**Histology**					1.000
IDC	37	95	188	90	
ILC	1	3	9	4	
Other	1	3	8	4	
na	0	0	4	2	
**Receptor status**					0.057
ER/PR +	24	62	155	74	
HER2 +	1	3	13	6	
TN	13	33	31	15	
na	1	3	10	5	
**Ki-67 index**					0.535
≥ 15%	32	82	152	73	
< 15%	2	5	21	10	
na	5	13	36	17	
**Grade**					0.214
1	3	8	27	13	
2	18	46	118	56	
3	14	36	54	26	
na	4	10	10	5	

Positive/negative refers to the presence/absence of a pathogenic or likely pathogenic variant (PV/LPV) in *BRCA1*, *BRCA2* or *TP53* genes. SD = standard deviation. na = not available. Localized: stages 0, I or IIA. Regional: stages IIB or III. Distant: stage IV. IDC: invasive ductal carcinoma. ILC: invasive lobular carcinoma. Fisher’s exact test was applied to all variables, except for analyses of age (where T-test was applied). All *p*-values < 0.05 are in bold.

### Variants identified in *BRCA1, BRCA2* and *TP53* genes

By combining sequencing methodologies (NGS and Sanger sequencing), 100% coverage of exonic and flanking intronic regions was achieved for the analyzed genes, with the intended quality (see Methods). All variants detected in *BRCA1*, *BRCA2* and *TP53* are listed in Supplementary Table S2. Thirty-four distinct pathogenic or likely pathogenic variants (PV/LPV) were identified in *BRCA1*, *BRCA2* and *TP53* in 41 of the 257 patients (15.9%), of which 17 PV/LPV were detected in *BRCA1* (20 patients), 14 PV/LPV in *BRCA2* (16 patients), and 3 PV/LPV in *TP53* (5 patients) (Fig. 1; Supplementary Fig. S1 and S2). Most of the 34 PV/LPV were nonsense substitutions (n = 11; 32.4%), followed by frameshift (n = 10; 29.4%), missense (n = 8; 23.5%), splicing-site variants (n = 4; 11.8%), and the *BRCA2* Alu insertion c.156_157insAlu (n = 1; 2.9%). Four variants classified as PV/LPV according to the ACMG/AMP criteria were not previously described in the literature (Supplementary Table S2), three in *BRCA1* (c.4925C > A, p.Ser1642Ter; c.5153-2A > C; c.5206G > T, p.Val1736Phe) and one in *BRCA2* (c.1849del, p.Ser617fs). The most frequently identified PV/LPV was *TP53* c.1010G > A (p.Arg337His), detected in three patients, followed by PV/LPV detected in two patients each: *BRCA1* c.441 + 2T > A, *BRCA1* c.4484G > T (p.Arg1495Met), *BRCA1* c.4964_4982del (p.Ser1655fs), *BRCA2* c.2T > G (p.Met1?), and *BRCA2* c.156_157insAlu. No double heterozygous patients carrying two PV/LPV were found.

Twenty-three distinct variants of uncertain significance (VUS) were identified in 27 of the 257 patients (10.5%), being 7 VUS in *BRCA1* (in 7 patients) and 16 VUS in *BRCA2* (in 20 patients), and no VUS was found in *TP53* (Supplementary Table S2, and Supplementary Fig. S1 and S2). Most of the 23 VUS were missense (n = 21; 91.3%), one was synonymous (4.3%) and one was intronic (4.3%). One of the missense VUS found in the *BRCA1* gene (c.3637G > A, p.Glu1213Lys) was not previously described in the literature. Supplementary Figure S2 shows that one *BRCA1* VUS is located in the BRCT domain (c.5348T > C, p.Met1783Thr) and that the six *BRCA2* VUS are dispersed throughout distinct motifs or domains of this gene (c.4616T > C, p.Leu1539Ser; c.5612G > A, p.Ser1871Asn; c.5986G > A, p.Ala1996Thr; c.7712A > G, p.Glu2571Gly; c.8122A > G, p.Thr2708Ala; c.8668C > A, p.Leu2890Ile). One patient presented a non-coding VUS in *BRCA1* (c.441 + 8C > T) concurring with one PV identified in the same gene (*BRCA1* c.1687C > T, p.Gln563Ter). One patient presented a VUS in *BRCA2* (c.2827A > G, p.Ile943Val) concurring with one PV identified in *ATM* gene (c.640del, p.Ser214fs). Apart from in silico prediction tools automatically employed by VarSome, all VUS were analyzed with prediction tools for splicing alteration. Eight of them were predicted to affect splicing by two or more prediction tools and received the PP3 ACMG/AMP criterion (Supplementary Table S2), but they remained classified as VUS.

### Clinicopathological data between carriers and non-carriers of PV/LPV

Comparisons between carriers and non-carriers of PV/LPV in *BRCA1*, *BRCA2* and *TP53* genes are detailed in Tables 1 and 2. Similar results were observed when considering PV/LPV only in *BRCA1* and *BRCA2* (Supplementary Tables S3 and S4). A significant difference was observed for laterality (Table 2), in that carriers of PV/LPV presented a significantly higher proportion of bilateral BC than non-carriers (*p* = 0.007, Fisher exact test). Considering *BRCA1* and *BRCA2* only (Supplementary Table S4), triple-negative tumors were more frequently observed in carriers than in non-carriers, while tumors positive for hormone receptors (ER/PR +) or HER2 + were less frequently found in carriers than in non-carriers (*p* = 0.027, Fisher exact test); receptor status results including carriers of PV/LPV in *TP53* are similar (Table 2), although not significant (*p* = 0.057, Fisher exact test).

**Figure 2:**
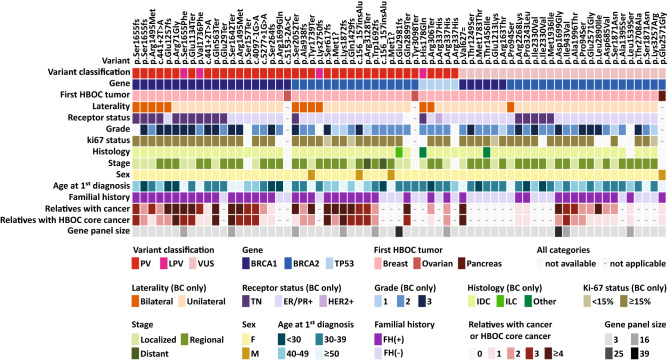
Clinicopathological characteristics and familial history of all carriers of PV, LPV or VUS in *BRCA1*, *BRCA2* or *TP53* genes. Each column represents the data of a single carrier, and columns are sorted by variant classification (PV/LPV × VUS), gene, HBOC first tumor, laterality, receptor status, relatives with cancer, and relatives with HBOC cancer. All carriers presented BC, OC or pancreatic cancer as first tumors; except the carrier of *BRCA1* VUS c.5348T > C, p.Met1783Thr, which presented thyroid cancer as first malignancy and BC as second. Carrier of p.Gln563Ter PV in *BRCA1* (11th column) also carries a non-coding VUS in *BRCA1* (c.441 + 8C > T). Gene panel size indicates if only *BRCA1*, *BRCA2* and *TP53* (= 3) were sequenced, or if the gene panel included other genes (totaling 16, 25 or 39 genes); but only 30 patients in the cohort were sequenced for genes beyond *BRCA1/2* and *TP53* (see Fig. 1). Carrier of p.Ile943Val VUS in *BRCA2* (55th column) is the only patient with PV/LPV or VUS in *BRCA1*, BRCA2 and *TP53* genes that also carries a PV/LPV in another gene (*ATM* PV c.640del, p.Ser214fs). See colored legend for details on each clinicopathological category. IDC: invasive ductal carcinoma. ILC: invasive lobular carcinoma. Localized: stages 0, I or IIA. Regional: stages IIB or III. Distant: stage IV.

Clinicopathological and familial data of all carriers of PV/LPV or VUS are summarized in Fig. 2. All 41 carriers of PV/LPV in *BRCA1*, *BRCA2* or *TP53* genes presented BC or OC as first tumors. Of the 16 patients that presented first tumors other than HBOC core tumors in the study cohort, none presented PV/LPV, and only one presented a VUS in *BRCA1* (c.5348T > C, p.Met1783Thr; see Fig. 2), with thyroid cancer as first malignancy and BC as the second.

### Familial history between carriers and non-carriers of PV/LPV

Table 1 shows that a total of 167/257 (65.0%) patients fulfilled NCCN criteria for familial history of HBOC (named FH(+) patients), of which 30 were carriers of a PV/LPV in *BRCA1/2* or *TP53* (30/167 = 18.0%), and 137 were non-carriers (137/167 = 82.0%). The remaining 90 patients in the cohort (90/257 = 35.0%) did not fulfill any NCCN criteria for familial history of HBOC (named FH(−) patients), and were not included in the following analyses; 11 of them were carriers of PV/LPV (11/90 = 12.2%).

Within the FH(+) group (Table 1), we observed that PV/LPV carriers had families with a significantly higher number of cancer cases (average of 3.6 relatives with cancer) than non-carriers (average of 2.8 relatives with cancer) (*p* = 0.024, Poisson regression). Regarding HBOC core tumors, FH(+) PV/LPV carriers also had a higher number of affected relatives than FH(+) non-carriers, albeit non-significant (*p* = 0.057, Poisson regression). The most recurrent HBOC core tumor in the relatives of FH(+) PV/LPV carriers was BC (75.3%), followed by prostate cancer (10.4%) and OC (9.1%) (Supplementary Fig. S3). But an effect of family size cannot be ruled out, since family size was also significantly higher in carriers (average of 25.4 relatives per family) than in non-carriers (average of 22.7 relatives per family) (Table 1; *p* = 0.004, Poisson regression); with a Spearman’s correlation of r_s_ = 0.25 (*p* = 0.001) between family size and the number of relatives with cancer.

Regarding non-HBOC core tumors, FH(+) PV/LPV carriers and FH(+) non-carriers presented the same frequency of affected relatives, 28.0% each (Supplementary Fig. S3), and no significant difference was observed when comparing the number of relatives with non-HBOC core tumors between these groups (Table 1; *p* = 0.232, Poisson regression). Supplementary Table S5 details the non-HBOC core tumors in relatives of FH(+) PV/LPV carriers or non-carriers. Only the proportion of relatives affected with hepatic cancer was significantly higher in FH(+) PV/LPV carriers than in FH(+) non-carriers (13% and 1%, respectively, *p* = 0.011, Poisson regression).

### Predictive power of risk models and familial history

**Figure 3:**
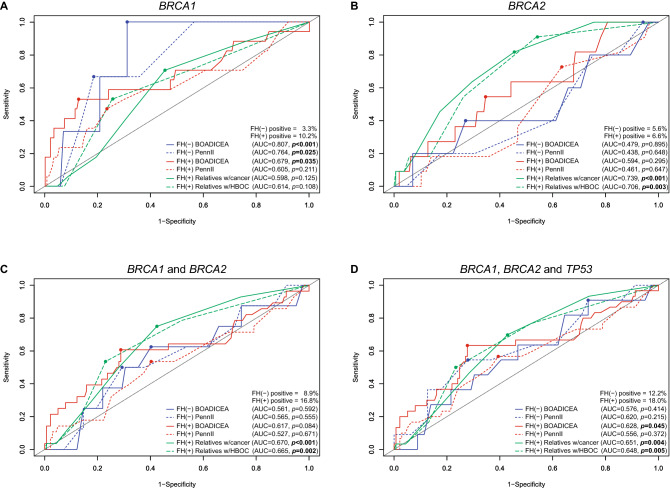
Predictive power of detecting PV/LPV carriers (performance) in (A) *BRCA1* only, (B) *BRCA2* only, (C) *BRCA1* and *BRCA2*, or (D) *BRCA1*, *BRCA2* and *TP53*, in two subgroups: FH(−) = patients with no familial history of cancer (n = 90); FH(+) = patients with familial history of cancer (n = 167). Performance was estimated through Receiver Operating Characteristic (ROC) curves for BOADICEA and Penn II risk prediction models, as well as for the familial history data (number of relatives with cancer). FH(−) positive or FH(+) positive refer to the percentage of PV/LPV cases in each subgroup. Relatives w/cancer refers to the number of relatives with cancer. Relatives w/HBOC refers to the number of relatives with HBOC core cancer, which was defined, for convenience, as breast, ovarian, pancreatic, and prostate cancer. The corresponding area under the ROC curve (AUC) and *p*-value are presented, as well as the optimal cutoff point (marked by a dot) estimated through the Youden index.

Since there are no universal cutoff values for BOADICEA and PennII prediction models, the predictive power of detecting PV/LPV carriers (performance) was estimated for each model using Receiver Operating Characteristic (ROC) curves (Supplementary Fig. S4). Regardless of the familial history, significant performance was observed for detecting PV/LPV carriers in *BRCA1* by both BOADICEA (AUC = 0.704, *p* = 0.004) and PennII (AUC = 0.651, *p* = 0.031), but no significant performance was observed for detecting PV/LPV carriers in *BRCA2* by any risk prediction model (AUC = 0.572, *p* = 0.353 for BOADICEA; and AUC = 0.459, *p* = 0.563 for PennII). The same pattern was observed when considering the subgroup of patients without familial history of cancer (FH(−)) (Fig. 3); in this case BOADICEA and PennII models were the best predictors of PV/LPV carriers in *BRCA1* (AUC = 0.807, *p* < 0.001; and AUC = 0.764, *p* = 0.025, respectively), but were poor predictors of PV/LPV carriers in *BRCA2* (AUC < 0.500, *p* > 0.05 in both cases).

In the subgroup of patients with familial history of cancer (FH(+)) (Fig. 3), when the number of relatives with cancer was used as a predictor of PV/LPV carriers it outperformed both prediction models for *BRCA2* (AUC = 0.739, *p* < 0.001), for *BRCA1/2* (AUC = 0.670, *p* < 0.001), and for *BRCA1/2* and *TP53* (AUC = 0.651, *p* = 0.004). Similar results were found when the number of relatives with HBOC core cancer was used as a predictor of PV/LPV carriers. In turn, BOADICEA was the best predictor of PV/LPV carriers in *BRCA1*, in the subgroup of FH(+) patients (AUC = 0.679, *p* = 0.035) (Fig. 3).

The optimal cutoff points for BOADICEA and PennII models varied between 7.0% and 20.9% in FH(+) patients, and between 1.6% and 8.0% in FH(−) patients, with no consistent value across all estimates (Supplementary Table S6). Considering *BRCA1* only, for which these prediction models presented their best performances, optimal cutoff points for BOADICEA and PennII models were, respectively, 20.9% and 11.0% in FH(+) patients, 3.7% and 8.0% in FH(−) patients, and 9.5% and 10.0% in the full cohort (FH(+ / −) patients). In contrast, the optimal cutoff point for the number of relatives with cancer as a predictor of PV/LPV carriers was estimated as three in all analyses. This result indicates that having three or more relatives with cancer was a consistent predictor of PV/LPV carriers in FH(+) patients in our cohort, especially for *BRCA2*. Similar results were observed when the number of relatives with HBOC core cancer was used as a predictor (Supplementary Table S6).

### Pathogenic or likely pathogenic variants identified in other genes

Of the thirty patients that were sequenced using MPS panels encompassing genes beyond *BRCA1*, *BRCA2* and *TP53* (see Fig. 1 and Materials and methods section), five presented PV/LPV as follows. Four of these patients presented PV/LPV in moderate-penetrance breast/ovarian cancer susceptibility genes (*ATM*, *BARD1*, *RAD51C*), while one patient presented a LPV in a gene with no established association with breast/ovarian cancer (*SLX4*), but for which loss-of-function variants might contribute to breast cancer in rare cases^33–35^. Three of these patients had FH(+) with 3 to 5 relatives with cancer (first- to third-degrees), and were diagnosed between 30 and 47 years of age: two patients presented BC as only tumor (one with PV in *ATM* gene: c.640del, p.Ser214fs; and one with PV in *BARD1* gene: c.176_177del, p.Glu59fs); and one patient presented BC as second tumor and melanoma as first and third tumors (LPV in *ATM* gene: c.9023G > A, p.Arg3008His). One patient that also had FH(+), with only one affected relative (third-degree with pancreatic cancer), presented BC as only tumor (diagnosed at 25 years of age), and carried a LPV in *SLX4* gene (c.4881del, p.Thr1628fs). Finally, one patient with FH(−) and with BC as the only tumor (diagnosed at 26 years of age) was found to have a non-coding spliceogenic LPV in RAD51C (c.965 + 5G > A).

## Discussion

In this study, we found that 41 out of 257 tested patients (15.9%) were carriers of germline PV/LPV in *BRCA1* (20 patients), *BRCA2* (16 patients) and *TP53* (5 patients) genes. Of the five *TP53* carriers, which were enrolled in this study based on NCCN criteria for HBOC, two did not fulfill any classic or Chompret criteria for Li-Fraumeni syndrome. Considering *BRCA1* and *BRCA2* only, prevalence of PV/LPV was 14.0%, which is within the range of the prevalence observed in other HBOC studies with Brazilian populations, when complete sequencing of *BRCA1/2* was performed (10.0–22.5%; see Guindalini et al.^36^). Besides, three deleterious variants found in *BRCA1* and one found in *BRCA2* were reported here for the first time, once again highlighting the heterogeneity of *BRCA1/2* variants in the Brazilian population^15,18,19,36^.

Our results indicate that including *TP53* in the setting of *BRCA1/2* testing might be cost-effective, since adding a single *TP53* multiplex PCR to a *BRCA1/2* assay resulted in the detection of four *TP53* PV/LPV carriers (Fig. 1), which represents 9.7% of all PV/LPV carriers in the present study. In fact, the most frequently identified deleterious variant in our cohort was *TP53* c.1010G > A (p.Arg337His), with three patients carrying this variant. *TP53* p.Arg337His is a Brazilian founder variant present in 0.3% of inhabitants from South and Southeast Brazilian regions, being less frequently reported in other Brazilian regions^21,36,37^. This variant is associated with a higher predisposition for cancers within the Li-Fraumeni Syndrome spectrum (BC, adrenocortical carcinoma, brain cancer, and gastric tumors), and is frequently reported among patients with hereditary BC in Brazil^21,36–41^, while the frequency of any germline *TP53* PV/LPV in other countries can be relatively low^1,42^. The high frequency of p.Arg337His emphasizes the importance of including *TP53* in routine testing of Brazilian patients suspected of hereditary BC or HBOC. Based on this accumulated evidence, Guindalini et al.^36^ discussed the need for differential guidelines for surveillance and risk-reduction strategies in patients with hereditary BC in Brazil, and our results contribute to this discussion.

Two other deleterious variants frequently reported in Brazilian patients were found in our cohort: *BRCA1* c.441 + 2T > A and the Alu element insertion *BRCA2* c.156_157insAlu; each detected in two patients. *BRCA2* c.156_157insAlu is a founder mutation from Portugal^43^, being the third most frequently reported *BRCA2* PV in Brazil^18^. *BRCA1* c.441 + 2T > A has been recurrently reported in studies with Brazilian patients, mainly from Southeast region^14,18,40,41,44,45^, with only one report from another country^46^, as far as we know. The apparent high frequency of *BRCA1* c.441 + 2T > A in Brazilian patients, including our study and two other HBOC patients from INCA’s Genetic Counseling Program that were enrolled before 2016 (unpublished data), raises the possibility that this variant might have experienced a founder effect in Brazil.

Interestingly, the *BRCA1* c.5266dupC (p.Gln1756fs) variant was not detected in our cohort. This is the most frequently reported *BRCA1* PV in Brazil, reaching up to 20% of PV/LPV variants in this gene^18^, and has a high frequency in HBOC patients from Central and East European populations^47^. Most reports of this variant in Brazil come from the South and Southeast regions^18^, where there is a major contribution of European ancestry to the population genetic background^48^. To our knowledge, only two other Brazilian studies, with patients from the Northeast region, did not report this variant^37,49^. In fact, *BRCA1* c.5266dupC (p.Gln1756fs) was reported in two previous studies with patients from our institution^50,51^, and by other studies with patients from Rio de Janeiro Estate^52^. The absence of *BRCA1* c.5266dupC in the 257 patients analyzed here may reflect some differences to the cohort of patients studied by Lourenço et al.^50^. We surmise that these differences could be related to changes in the socio-economic profile of patients assisted by our institution (INCA), with the implementation of new management practices by the Brazilian public healthcare system^53^.

Regarding the variants classified as VUS, 10.5% of the 257 patients presented VUS in *BRCA1/2*, with no VUS detected in *TP53*. Two VUS called our attention due to the familial history of cancer of the proband: carrier of *BRCA1* c.1881C > G (p.Val627 =) reported six relatives diagnosed with HBOC core tumors; while the carrier of *BRCA2* c.5096A > G (p.Asp1699Gly) reported eight relatives diagnosed with cancer, but only two with HBOC core tumors. Nevertheless, these VUS are located in exon 11 of each gene, which are both estimated to be coldspots of pathogenic variants^54^. Three other VUS called our attention due to early-onset of cancer in the proband, as follows. Carrier of *BRCA1* c.5348T > C (p.Met1783Thr) in exon 22 was diagnosed with thyroid cancer at 14 years of age and with BC at 27. This variant is located in the BRCT domain, which is a critical domain where missense variants can lead to loss of BRCA1 function^54^, but functional studies have presented conflicting results^55,56^. Carrier of *BRCA2* c.9728C > T (p.Pro3243Leu) in exon 27 and carrier of *BRCA2* c.6988A > G (p.Ile2330Val) in exon 13 were diagnosed with triple-negative BC at 23 and 24 years old, respectively, even though another carrier of c.6988A > G (p.Ile2330Val) was diagnosed with triple-negative BC at a later onset age (49 years old). Among these VUS, only c.9728C > T (p.Pro3243Leu) was predicted to be spliceogenic (Supplementary Table S2). We note that 12 out of 21 (57.1%) VUS identified in the present work (Supplementary Table S2) are located in exon 11 of *BRCA1* or *BRCA2*, which are predicted to be coldspot regions where pathogenic variants are unlikely occur^54^. We also note that specific guidelines for *BRCA1* and *BRCA2* variant classification are yet to be defined by the ENIGMA Variant Curation Expert Panel^57^, and will aid the reclassification of VUS in the future. Further segregation analysis or functional studies might also contribute to the classification of pathogenicity of these variants.

Two clinicopathological features were significantly associated with mutational status: laterality and receptor status. The association with bilateral breast cancer reflects an increased risk of developing contralateral BC in carriers of PV/LPV in *BRCA1/2* genes. Kuchenbaecker et al.^7^ reported that the cumulative risk for contralateral BC is 40% for *BRCA1* PV/LPV carriers and 26% for *BRCA2* PV/LPV carriers, 20 years after first BC diagnosis. In respect to receptor status, our findings were similar to Mavaddat et al.^58^, which also found a significant association of triple-negative BC tumors and the presence of PV/LPV in *BRCA1* or *BRCA2* genes.

Concerning the familial history of cancer, we observed that families of PV/LPV carriers had a significantly higher proportion of cancer cases than families of non-carriers, but an effect of family size could not be ruled out. The most recurrent HBOC core tumor in the relatives of FH(+) PV/LPV carriers was BC, followed by prostate cancer and OC, although no significant difference was found for the number of relatives with HBOC core cancer between carriers and non-carriers of PV/LPV. Of note, when analyzing non-HBOC core tumors, a significantly higher proportion of hepatic cancer was found in relatives of *BRCA1/2* PV/LPV carriers than in non-carriers, suggesting that hepatic cancer could be a recurrent phenotype in the cancer spectrum of HBOC families. Supporting this suggestion, other authors also reported a higher proportion of hepatic cancer in families of *BRCA1/2* PV/LPV carriers, as well as a higher proportion of gallbladder and gastric cancer^59–62^.

Some limitations of the present work need to be addressed. To date, hereditary cancer risk assessment is not systematically assessed in the Brazilian public Unified Health System (SUS). At the Brazilian National Cancer Institute (INCA), referral to the Genetic Counseling Program might still be biased towards individuals diagnosed with BC, which may result in under-reporting of other HBOC spectrum cases and associated germline variants. We were also limited by reliance on the family histories provided by probands and/or relatives, which are not always complete. Another limitation is that only 5/257 (1.9%) of the patients were tested for large rearrangements in *BRCA1/2* (at private laboratories; all five with negative results). And, only 30 patients were sequenced for HBOC related-genes beyond *BRCA1/2* and *TP53*, five of which presented PV/LPV in *ATM*, *BARD1*, *RAD51C* and *SLX4* genes (see Fig. 1 and Materials and methods section); but prevalence of PV/LPV in other HBOC related-genes cannot be estimated due to a possible bias in the sub-selection of tested patients. Therefore, it is possible that PV/LPV related to large rearrangements in *BRCA1/2* and *TP53* genes or to other HBOC related genes could still be undetected in our cohort.

Risk prediction models for *BRCA1/2* have been extensively used and validated in European and North American populations^63,64^, but less evaluated in other populations^65^. According to NCCN guidelines^30^, individuals presenting prior probability of carrying *BRCA1/2* PV/LPV greater than 5% should be eligible for genetic testing. In the present cohort of Brazilian patients suspected of HBOC, BOADICEA and PennII models showed significant performances for estimating the risk of carrying PV/LPV in *BRCA1*, but poor performances for estimating the risk carrying PV/LPV in *BRCA2*. Even for *BRCA1*, no consistent optimal cutoff of prior probability values was observed for these models, ranging from 3.7% to 20.9% across the subgroups of patients with (FH(+)) or without (FH(-)) NCCN criteria for familial history, while averaging from 9.5% to 10.0% in the full cohort (FH(+ / −) patients). In fact, BOADICEA and PennII models tend to perform worse for *BRCA2* than for *BRCA1*, regardless of the population^65–67^. And it is not surprising that prediction models might not perform at their best for *BRCA1* and *BRCA2* in populations or ethnic groups that have genetic backgrounds distinct from those used in the development and validation of these models^66,68,69^, as is the case for the Brazilian population^48^.

In turn, when the familial history of cancer was used as a predictor of PV/LPV carriers, it outperformed BOADICEA and PennII for *BRCA2*, for *BRCA1/2*, and for *BRCA1/2* plus *TP53*; similar results were observed when the number of relatives with HBOC core cancer was used as a predictor. The optimal cutoff point for the number of relatives with cancer was estimated as three in all analyses. This result indicates that patients suspected of HBOC presenting three or more relatives with cancer could be at greater risk of carrying PV/LPV in the analyzed genes; we note that any undetermined or possible environmentally-related cancers, like respiratory system tumors, were excluded from FH(+) analyses. Even though a correlation between the number of relatives with cancer and family size cannot be disregarded in our cohort, the performance of familial history in risk prediction for PV/LPV in *BRCA1/2* genes might be of importance in the Brazilian population. Alemar et al.^70^ analyzed 54 international testing criteria (including NCCN) and mutation probability algorithms (including PennII model) in a cohort of Southern Brazilian patients that fulfilled NCCN HBOC testing criteria. Of the 25 criteria that reached statistically significant odds-ratio of carrying a PV/LPV in *BRCA1/2*, nine (36%) were exclusively related to the familial history of probands^70^. Thinking forward to a scenario where HBOC genetic testing could be implemented in the Brazilian public healthcare system, as well as in mid- to low-income countries, limited resources would still require prioritization of patients at high-risk. In this scenario, identification of the most informative criteria for each population is essential, as discussed by Alemar et al.^70^. In our full cohort, patients were at greater risk of carrying PV/LPV when risk prediction estimates were greater than 10.0% (for *BRCA1*) or when presenting three or more relatives with cancer (for *BRCA1* and *BRCA2*). Further studies, with greater and more diverse representativeness of Brazilian population, could provide an optimized strategy for identification of Brazilian HBOC patients at high-risk of carrying PV/LPV in *BRCA1/2* and *TP53*, through a combination of international guidelines, risk prediction models (especially for *BRCA1*), and familial history of cancer (especially for *BRCA2*).

## Material and methods

### Patients and clinical data

Patients of the Brazilian National Cancer Institute (INCA) with current or prior cancer diagnosis that were also suspected of HBOC and were referred to the Genetic Counseling Program, from February 2016 to August 2019, were eligible for this study. A total of 265 patients that fulfilled at least one criterion of the National Comprehensive Cancer Network Guidelines^32^ for personal or familial high-risk assessment of breast and ovarian cancer were considered for this study. However, eight patients were excluded because: diagnosis of cancer in the HBOC spectrum was not confirmed (four patients); three patients performed molecular testing at private laboratories, but results were not provided; and one patient was a PV carrier in *MSH2* gene, previously reported in a relative. Hence, 257 patients were maintained in this study (Fig. 1). All patients signed an informed consent, and all procedures were approved by the institutional research ethics committee (projects: INCA 114/07 and CAAE 14144819.0.0000.5274).

Patient clinicopathological data and familial history of cancer were collected by institutional certified medical geneticists. The collected data included: date of birth, sex, medical records, cancer diagnosis by primary site, age at cancer diagnosis, cancer laterality (when appropriate), personal history of other primary cancers (tumor types and location), histological subtype, tumor immunohistochemical profile (for BC cases), and familial history of cancer. Patient cancer staging and hormonal receptor status were obtained from the medical records. Cancer staging was reported based on the TNM classification system of the Union for International Cancer Control (https://www.uicc.org/resources/tnm). For breast tumors, histological types and grades were annotated, respectively, as recommended by the World Health Organization International Classification of Diseases for Oncology and by the Nottingham Grading System. Breast tumors were classified as: triple-negative (TN) if negative for estrogen (ER), progesterone (PR) and HER2; as HER2 positive (HER2 +) if negative for ER, PR and positive for HER2; and as hormone receptor positive (ER/PR +) if positive for ER and/or PR, regardless of HER2 status. ER and PR were considered positive if > 1% of the examined cells stained for the respective receptor, while HER2 was considered positive if the expression was scored as 3 + or if HER2 amplification was detected by FISH^71^. The Ki-67 index of cell proliferation was considered high if ≥ 15% of the examined cells were stained for Ki-67 nuclear protein^72^.

### DNA isolation, multiplex PCR and NGS library preparation

Genomic DNA was isolated from peripheral blood samples using salting out method^73^. Multiplex PCR-based assays were designed and performed as described by Gomes et al.^17^; *TP53* findings for 67 patients from the present study (out of 257) were published in this previous study^17^. Briefly, PCR primers were designed to amplify the full coding regions of *BRCA1* (NG_005905.2), *BRCA2* (NG_012772.3) and *TP53* (NG_017013.2), including at least 12 bp of intronic regions flanking the 5’ and 3’ ends of each exon; except for the 3’ end of exon 9 from *TP53*, where only 9 bp of flanking intronic region was included. Primer pairs were pooled in thirteen distinct multiplex PCR-based assays, which were performed using Platinum Taq DNA Polymerase (Invitrogen): six multiplex assays for *BRCA1*, six for *BRCA2*, and a single assay for *TP53*. Multiplex PCR products were purified using PureLink PCR purification kit (Invitrogen), quantified on a NanoDrop100 spectrophotometer (ThermoFisher), and pooled together for each sample, using a rough normalization of base pair counts per multiplex PCR. Primer pairs, multiplex PCR conditions, and pooling ratios are available under request. Final pool of multiplex PCR products from each patient was quantified on a Qubit 3.0 Fluorometer (Invitrogen) using High Sensitivity (HS) assay kit (Invitrogen). Sequencing libraries were prepared using Nextera XT DNA kit (Illumina), following manufacturer’s instructions.

### Massive parallel sequencing (MPS) and data analysis

Libraries were paired-end sequenced (2 × 150 cycles) on a HiSeq2500 Genome Analyzer instrument (Illumina). Raw sequencing reads (FASTQ files) were mapped against the human reference genome sequence (GRCh37/hg19) using Burrows–Wheeler Alignment^74^ with default parameters. The Picard package was used to convert the sequence alignment map (SAM) files into BAM format, and to remove duplicate reads. Sorting and indexing procedures were undertaken with Samtools, while base quality score recalibration, indel realignment, and variant calling were performed using a pipeline based on the best practices of Genome Analysis Toolkit v4.1 (GATK) (Broad Institute). Variants were filtered out if depth mean coverage was < 27X, call quality (Q score) < 30, and if the variant allele frequency (VAF) was ≤ 20%, based on the theoretical model proposed by Leeneer et al.^75^. Sequence variants were annotated using Variant Effect Prediction^76^.

To evaluate the efficiency and accuracy of this MPS assay (multiplex PCR-based assays, MPS, and pipeline analysis), we re-sequenced 24 samples of patients that were enrolled in the Genetic Counseling Program before 2016 and had complete Sanger sequencing results for *BRCA1* and *BRCA2* genes. Of the 251 variants previously identified by Sanger sequencing in these 24 samples, 238 variants were also detected by our MPS assay and pipeline analysis. The overall sensitivity, positive predictive value (PPV), and false discovery rate (FDR) for *BRCA1/2* were estimated as 97.5%, 97.4% and 2.0%, respectively. Of the 13 variants not identified by our MPS assay, 12 were located in regions with low sequencing coverage (average depth coverage < 27X), and the remaining was the *BRCA2* c.156_157insAlu mutation (an Alu insertion in exon 3 of *BRCA2*), detected by an independent assay based on nested-PCR and Sanger sequencing; see next section.

### Sanger sequencing and identification of *BRCA2* c.156_157insAlu

In order to cover the sequencing gaps for all regions with average depth coverage < 27X in the MPS assays, and to confirm the presence of all MPS variants classified as pathogenic, likely pathogenic or VUS, single PCR assays followed by Sanger sequencing were performed on an ABI 3730 sequencer (Applied Biosystems). Primer pairs and PCR conditions are available under request. All PCR products were purified using PureLink PCR purification kit (Invitrogen). Cycle sequencing was performed using BigDye® Terminator kit version 3.1 (ThermoFisher) according to the manufacturer’s instructions. SeqMan (Lasergene version 8; DNASTAR) and novoSNP^77^ were used for sequence alignment and visualization of Sanger sequencing data. For all samples, the presence of *BRCA*2 c.156_157insAlu mutation in exon 3 of *BRCA*2 was assessed through nested PCR followed by Sanger sequencing, as described by Peixoto et al*.*^43^.

### Other MPS methodologies

Alternative MPS methodologies were employed for 38 of the 257 patients (Fig. 1), which were selected due to convenience at the time in which these panels were available; the classification of each PV/LPV detected in these patients was confirmed as described in the next section.

For 16 patients, MPS data were obtained through Ion AmpliSeq Customized Panel (ThermoFisher), which covers whole exons and splicing sites of genes *ATM*, *BARD1*, *BRCA1*, *BRCA2*, *BRIP1*, *CDH1*, *CHEK2*, *MRE11A*, *MUTYH*, *NBN*, *PALB2*, *PTEN*, *RAD50*, *RAD51C*, *STK11* and *TP53*. Genomic libraries were prepared as described in Coutinho et al.^78^, library sequencing was performed with Ion Torrent Personal Genome Machine™ (PGM) (ThermoFisher). For six patients MPS data were obtained through Multiplicom Agilent SureMastr HC panel, which covers whole exons and splicing sites of genes *ATM*, *BARD1*, *BLM*, *BRCA1*, *BRCA2*, *BRIP1*, *CDH1*, *CHEK2*, *FAM175A*, *MEN1*, *MLH1*, *MRE11A*, *MSH2*, *MSH6*, *MUTYH*, *NBN*, *PALB2*, *PMS2*, *PTEN*, *RAD50*, *RAD51C*, *RAD51D*, *STK11*, *TP53*, *XRCC2*, and 3’ UTR of *EPCAM*. Genomic libraries were prepared following the manufacturer's protocol, library sequencing was performed with MiSeq (Illumina). Data of 16 other patients were obtained through MPS performed at private laboratories (see Fig. 1): eight patients were sequenced with panels of 16 to 207 genes that included *BRCA1/2* and *TP53* (gene panel lists are available under request, two of which include the *SLX4* gene); and eight patients were sequenced only for *BRCA1/2*, while *TP53* was later sequenced through Sanger sequencing in our laboratory, except for one patient because DNA sample was not available. Private laboratories did not report likely benign and benign variants.

### Variants classification

The Human Genome Variation Society (HGVS) guidelines were used to describe the detected variants^79^, see Supplementary Table S2. We followed the guidelines of the American College of Medical Genetics and Genomics (ACMG) and the Association for Molecular Pathology (AMP) for classification and interpretation of clinical significance of the germline variants^80^ using the VarSome variant search engine version 8.4.6 as a guide^81^. Each automated ACMG/AMP criterion used by VarSome was carefully revised, as thoroughly described by Gomes et al.^17^ and in the legend of Supplementary Table S2 (see the Supplementary Information S1). Additional databases assessed for variant classification were ClinVar (https://www.ncbi.nlm.nih.gov/clinvar/) and gnomAD (gnomAD v2.1.1). To further evaluate PP3 or BP4 ACMG/AMP criteria, regarding potential splicing effects, all variants classified as VUS were also analyzed by the following prediction tools: ESRseq^82^ and HEXplorer^83^, which predict if the tested variants could alter splicing regulatory elements (SREs), and SpliceAI^84^, a deep neural network that predicts if the tested variants could create cryptic splicing sites.

### Statistical analysis

Patients were grouped as positive or negative regarding the presence or absence of a pathogenic or likely pathogenic variant (PV/LPV) in *BRCA1*, *BRCA2* or *TP53* genes. Personal and clinical features were compared between positive (PV/LPV carriers) and negative (non-carriers) groups: sex, age at diagnosis, and types of tumors for all patients; laterality, tumor stage, histological types, receptor status, Ki-67 index, and tumor grade for breast cancer patients only. Comparison of quantitative variables were carried out with the T-Test, while Fisher’s Exact Test was used for qualitative variables; *p*-values < 0.05 were considered statistically significant. All analyses, including Poisson Regression (see below), were carried out with SPSS version 26.0.0.0 software.

Familial history of cancer (named FH(+)) was analyzed for 167 patients, for which one side (146 patients) or both sides (21 patients) of the family fulfilled any NCCN criteria for familial history of HBOC. Considering the given side (or sides), family size and number of relatives with cancer were compared between PV/LPV carriers and non-carriers groups for the 167 patients with FH(+), using a Poisson Regression, as performed by Maves et al.^85^. To reduce potential bias from undetermined or possible environmentally-related cancers, regardless of patient mutational status, 162 familial cancer cases were excluded from these analyses: 68 cancer cases reported as undetermined, 55 cancer cases reported generically as female reproductive system, and 39 cases reported as cancers associated with respiratory system. Patients that did not fulfill any NCCN criteria for familial history of HBOC (named FH(−)) were not included in these analyses.

Based on personal and familial history information, BOADICEA^86^ and PennII model^31,67^ were used to predict the pre-test probability (prior probability) of harboring pathogenic variants in *BRCA1/2* genes, even though no cutoff value was used as inclusion/exclusion criteria. The predictive power of detecting PV/LPV carriers was estimated for each model, as well as for the familial history data (FH(+)), through receiver operating characteristic (ROC) curves using easyROC^87^.

### Statement

The authors declare that all methods were carried out in accordance with relevant guidelines and regulations.

### Patient consent

All individuals who participate in this study provided written informed consent approved by the local ethics committee. No identifiable personal patient data are shown in this article.

### Research involving human rights

All procedures performed in studies involving human participants were approved by the ethical committee of the Brazilian National Cancer Institute (INCA; projects #114/07 and CAAE 14144819.0.0000.5274).

## Supplementary Information

Supplementary Information 1.

Supplementary Information 2.

## Data Availability

All genetic variants described in the current study are available in ClinVar, SUB11475847, regardless of the sequencing methodology. Raw NGS data generated during this study are available in the DDBJ Center, Submission DRA014261, Bioproject PRJDB13663 with BioSamples SAMD00491063 to SAMD00491294 for 231 out of the 257 analyzed samples. The remaining samples were sequenced at private laboratories (raw data are not available) and/or by Sanger sequencing (raw data are available upon request to the corresponding author A.C.E.S.). For sample Matta2022_007, the vcf file is available upon request to A.C.E.S. Clinicopathological and familial data of each patient that participated in this study are not available, since these data cannot be shared publicly, due to ethical restrictions.
