# Supplementary material for: Familial history and prevalence of BRCA1, BRCA2 and TP53 pathogenic variants in HBOC Brazilian patients from a public healthcare service
Source: Sci Rep. 2022 Nov 3;12:18629. doi: 10.1038/s41598-022-23012-3 (PMC9633799; doi:10.1038/s41598-022-23012-3)
Supplement: Supplementary file 1 — Supplementary Information 1. [file 41598_2022_23012_MOESM1_ESM.pdf]

Familial history and prevalence of *BRCA1*, *BRCA2* and *TP53* pathogenic variants in HBOC Brazilian patients from a public healthcare service (Supplementary Information)

Authors: Bruna Palma Matta, Renan Gomes, Daniel Mattos, Renata Olicio, Caroline Macedo Nascimento, Gerson Moura Ferreira, Ayslan Castro Brant, Mariana Boroni, Carolina Furtado, Valdirene Lima, Miguel Ângelo Martins Moreira, Anna Cláudia Evangelista dos Santos.

Supplementary Table S1. Summary of personal history of cancer in the index patients.

Cancer type of index patients	1st cancer	2nd cancer	3rd cancer	4th cancer
Breast	232	46	1	1
Ovarian	7	2	0	0
Pancreatic	2	0	0	0
Melanoma	3	1	2	1
Lymphoma	4	0	0	0
Colorectal	3	1	3	0
Thyroid	2	1	0	0
Endometrial	2	0	0	0
Uterine	1	0	0	0
Gastrointestinal	1	3	0	0
Renal	0	1	0	0
Glioblastoma	0	1	0	0
Skin (non-melanoma)	0	0	1	2
Lung	0	0	1	0
**TOTAL**	257	56	8	4
**Average age at HBOC core cancer * Mean (SD)**	41.1 (11.7)	48.3 (13.7)	28.0 (0.0)	74.0 (0.0)
**Average age at other cancer Mean (SD)**	41.9 (15.4)	53.3 (14.8)	57.3 (10.7)	55.0 (12.7)

* HBOC core cancer refers to breast, ovarian, prostate, and pancreatic cancer; but no prostate cancer was observed in the index patients.

Supplementary Table S2 (see the separate excel file). List of all variants detected in *BRCA1*, *BRCA2* and *TP53* genes, including variant annotation, ACMG/AMP criteria, gnomAD allele frequencies (non-cancer database v2.1.1), ClinVar classification, prediction scores, and spliceogenic predictions of variants classified as VUS. We followed the guidelines of ACMG/AMP (PMID: 25741868) for variant classification, using the VarSome variant search engine version 8.4.6 as a guide (PMID: 30376034). And each automated ACMG/AMP criterion used by VarSome was carefully revised, as follows. ACMG/AMP PVS1 criterion was revised as specified by ClinGen Sequence Variant Interpretation (SVI) Recommendation (PMID: 30192042). ACMG/AMP criteria for population data (BA1, BS1, BS2 and PM2), case-control data (PS4), reputable source (PP5 and BP6), and in silico predictions (PP3, BP4 and BP7) were revised following CanVIG-UK (PMID: 32170000) Consensus Specification for Cancer Susceptibility Genes (CanVIG-UK CSGs v2.16: https://www.cangene-canvaruk.org/canvig-uk-guidance). BA1 (≥1%), BS1 (≥0.1%) and PM2 (absent or ≤0.002%) were applied to the maximum allele frequency in any gnomAD continental population. BS2 (≥3 homozygotes) was applied to the maximum homozygote count in any gnomAD continental population, except when BA1 was already awarded. PM2 was applied at supporting level only (PM2_Supporting), and variants awarded PVS1 + PM2_Supporting were classified as likely pathogenic, following ClinGen SVI Recommendation for PM2 v1.0 (https://www.clinicalgenome.org/working-groups/sequence-variant-interpretation/). Besides, PM2_Supporting was not counted as contrasting evidence to pull down a likely benign classification into VUS. Case-control data (PS4) was applied following CanVIG-UK CSGs v2.16 recommendations: case-control analysis with NHSD data, and case counting approach, for which we used PS4≥5 different families with HBOC criteria (PMID: 24936644, 29907814, 28490613, 32039725). Reputable source criteria were applied to ClinVar classification after 2018 (PP5 for LP/P; BP6 for LB/B), provided that the classification was approved by ClinGen Expert Groups (3 stars on ClinVar) or was given by ≥2 accredited commercial diagnostics laboratories (see CanVIG-UK CSGs v2.16). Regarding in silico predictions, REVEL scores were used for missense variants (PP3>0.7; BP4<0.4), and SpliceAI masked delta scores were used for synonymous and splicing-site/intronic variants (PP3≥0.2; BP4/BP7<0.2); see CanVIG-UK CSGs v2.16. We further used VarSome ratio of deleterious:benign predictions (PP3≥2:1 ratio; BP4≤1:2 ratio) to variants with REVEL score between 0.4-0.7 or no REVEL score. And PP3 was awarded to any variant classified as VUS when presenting ≥2 deleterious predictions in splicing alteration algorithms, regardless of REVEL score or VarSome ratio. As for criteria combination, PP3 was not awarded when PVS1 was applied (see CanVIG-UK CSGs v2.16), but PP3 was used as contrasting evidence towards a VUS classification. We note that exon 11 of *BRCA1* and exons 10-11 of *BRCA2* are estimated to be coldspots of pathogenic variants (PMID: 31911673), so that missense VUS located within these exons are unlikely to be pathogenic and will probably be reclassified in the future. The upcoming *BRCA1/2* variant classification guideline is also expected to aid in the reclassification of *BRCA1/2* VUS (ClinGen ENIGMA *BRCA1* and *BRCA2* Variant Curation Expert Panel: https://clinicalgenome.org/affiliation/50087/).

Supplementary Table S3. Study cohort; clinical and familial data according to mutational status of *BRCA1/2* only (excluding *TP53* results)

Clinical data	**Positive (n=36)**	**%**	**Negative (n=221)**	**%**	***p***
Sex					0.117
Female	33	92%	215	97%	
Male	3	8%	6	3%	
First HBOC core cancer					0.452
Breast	34	94%	214	97%	
Ovarian	2	6%	5	2%	
Pancreatic	0	0%	2	1%	
Multiple tumors					0.192
Yes	11	31%	45	20%	
No	25	69%	176	80%	
Age at first HBOC core cancer					0.860
< 30	6	17%	25	11%	
30 - 39	13	36%	90	41%	
40 - 49	8	22%	51	23%	
≥50	9	25%	55	25%	
Mean (SD)	41.3 (11.7)		41.7 (12.2)		
Median	39		39		
Familial data of FH(+) patients*	**Positive (n=28)**		**Negative (n=139)**		***p***
Relatives with cancer Mean (SD)	3.7 (1.7)		2.8 (1.8)		**0.011**
Relatives with HBOC core cancer Mean (SD)	2.6 (1.4)		2.0 (1.3)		**0.033**
Relatives with Other cancer Mean (SD)	1.0 (1.2)		0.8 (1.3)		0.169
Family size Mean (SD)	26.7 (16.7)		22.4 (12.2)		**<0.001**

Positive/negative refers to the presence/absence of a pathogenic or likely pathogenic variant (PV/LPV) in *BRCA1* or *BRCA2* genes. SD = standard deviation. For convenience, HBOC core cancer refers to breast, ovarian, pancreatic, and prostate cancer; but no prostate cancer was observed in the index patients. * Familial data of patients with FH(+) excludes 90 patients (FH(−)) that did not fulfill any NCCN criteria for familial history of HBOC. Fisher's exact test was applied to all variables, except for analyses of age (where T-test was applied), and familial data (where Poisson regression was applied). All *p*-values <0.05 are in bold.

Supplementary Table S4. Breast cancer patients; clinical data according to mutational status of *BRCA1/2* only (excluding *TP53* results) in these patients.

Clinical data	**Positive (n=34)**	**%**	**Negative (n=214)**	**%**	***p***
Sex					0.081
Female	31	91%	209	98%	
Male	3	9%	5	2%	
Age at first BC					0.621
<30	6	18%	22	10%	
30 - 39	12	35%	88	41%	
40 - 49	8	24%	50	23%	
≥50	8	24%	54	25%	
Mean (SD)	40.5 (11.4)		41.9 (12.0)		
Median	39		39		
Laterality					**0.017**
Unilateral	25	74%	191	89%	
Bilateral	9	26%	23	11%	
Stage					0.377
Localized	11	32%	85	40%	
Regional	20	59%	120	56%	
Distant	2	6%	5	2%	
na	1	3%	4	2%	
Histology					0.764
IDC	33	97%	192	90%	
ILC	1	3%	9	4%	
Other	0	0%	9	4%	
na	0	0%	4	2%	
Receptor status					**0.027**
ER/PR+	21	62%	158	74%	
HER2+	0	0%	14	7%	
TN	12	35%	32	15%	
na	1	3%	10	5%	
Ki-67 index					0.328
≥15%	29	85%	155	72%	
<15%	2	6%	21	10%	
na	3	9%	38	18%	
Grade					0.144
1	3	9%	27	13%	
2	14	41%	122	57%	
3	14	41%	54	25%	
na	3	9%	11	5%	

Positive/negative refers to the presence/absence of a pathogenic or likely pathogenic variant (PV/LPV) in *BRCA1* or *BRCA2* genes. SD = standard deviation. na = not available. Localized: stages 0, I or IIA. Regional: stages IIB or III. Distant: stage IV. IDC: invasive ductal carcinoma. ILC: invasive lobular carcinoma. Fisher's exact test was applied to all variables, except for analyses of age (where T-test was applied). All *p*-values <0.05 are in bold.

Supplementary Table S5. Cancer types in relatives with non-HBOC core tumors ("relatives with other cancer" in Table 1), according to the mutational status of *BRCA1, BRCA2* and *TP53* genes of their index patients (FH(+) only).

**Tumor Type**	**Positive (n=30)**	**%**	**Negative (n=137)**	**%**	***p*-value**
Abdomen (non-specified)	0	0%	3	2%	-
Esophageal	1	3%	7	5%	0.689
Gastric	4	13%	18	13%	0.979
Colorectal	5	17%	21	15%	0.866
Hepatic	4	13%	2	1%	**0.011**
Gallbladder	1	3%	0	0%	-
Bladder	0	0%	3	2%	-
Renal	0	0%	2	1%	-
Testicular	0	0%	1	1%	-
Head and neck	3	10%	21	15%	0.489
Central Nervous System	5	17%	8	6%	0.066
Hematologic	3	10%	9	7%	0.529
Bone	1	3%	2	1%	0.500
Skin (non-melanoma)	1	3%	3	2%	0.716
Endocrine	2	7%	7	5%	0.740

Data refers to cancer types of relatives with non-HBOC core cancer ("relatives with other cancer" in Table 1) grouped by the mutational status of *BRCA1*, *BRCA2* and *TP53* genes of their index patients (FH(+) only): positive/negative refers to the presence/absence of a pathogenic or likely pathogenic variant (PV/LPV) in *BRCA1*, *BRCA2* or *TP53*; n refers to the number of FH(+) index patients in each group. Familial data of patients with FH(+) excludes 90 patients (FH(−)) that did not fulfill any NCCN criteria for familial history of HBOC. Non-HBOC core cancer excludes breast, ovarian, pancreatic, and prostate cancer. Poisson regression was applied to all cases; all *p*-values <0.05 are in bold.

Supplementary Table S6. Predictive power of detecting PV/LPV carriers (performance), estimated through Receiver Operating Characteristic (ROC) curves for BOADICEA and PennII risk prediction models, and for familial history (number of relatives with cancer or HBOC core cancer) in FH(+) patients.

Familial history	Gene	Predictor	AUC ± SE	95% CI	z-value	*p*-value	Optimal cutoff
FH(+) (n=167)	*BRCA1*	BOADICEA	0.679 ± 0.085	0.513 - 0.845	2.109	**0.035**	20.9%
		PennII	0.605 ± 0.084	0.441 - 0.769	1.251	0.211	11.0%
		Relatives with cancer	0.598 ± 0.064	0.473 - 0.724	1.535	0.125	3
		Relatives with HBOC core cancer	0.614 ± 0.071	0.475 - 0.753	1.606	0.108	3
	BRCA2	BOADICEA	0.594 ± 0.090	0.418 - 0.770	1.047	0.295	9.8%
		PennII	0.461 ± 0.085	0.294 - 0.628	-0.457	0.647	7.0%
		Relatives with cancer	0.739 ± 0.066	0.609 - 0.869	3.614	**<0.001**	3
		Relatives with HBOC core cancer	0.706 ± 0.069	0.571 - 0.841	2.998	**0.003**	2
	BRCA1/2	BOADICEA	0.617 ± 0.068	0.484 - 0.749	1.729	0.084	9.8%
		PennII	0.527 ± 0.064	0.402 - 0.653	0.425	0.671	8.5%
		Relatives with cancer	0.670 ± 0.051	0.571 - 0.769	3.363	**0.001**	3
		Relatives with HBOC core cancer	0.665 ± 0.053	0.561 - 0.770	3.111	**0.002**	3
	BRCA1/2 and *TP53*	BOADICEA	0.628 ± 0.064	0.503 - 0.753	2.006	**0.045**	9.8%
		PennII	0.556 ± 0.063	0.433 - 0.678	0.893	0.372	8.5%
		Relatives with cancer	0.651 ± 0.050	0.553 - 0.748	3.038	**0.002**	3
		Relatives with HBOC core cancer	0.648 ± 0.052	0.546 - 0.751	2.830	**0.005**	3
FH(−) (n=90)	*BRCA1*	BOADICEA	0.807 ± 0.079	0.651 - 0.962	3.860	**0.000**	3.7%
		PennII	0.764 ± 0.118	0.533 - 0.996	2.240	**0.025**	8.0%
	BRCA2	BOADICEA	0.479 ± 0.161	0.164 - 0.794	-0.132	0.895	4.0%
		PennII	0.438 ± 0.137	0.170 - 0.705	-0.457	0.648	4.0%
	BRCA1/2	BOADICEA	0.561 ± 0.114	0.338 - 0.784	0.536	0.592	3.6%
		PennII	0.565 ± 0.110	0.350 - 0.780	0.590	0.555	7.5%
	BRCA1/2 and *TP53*	BOADICEA	0.576 ± 0.093	0.394 - 0.758	0.816	0.414	1.6%
		PennII	0.620 ± 0.097	0.430 - 0.810	1.241	0.215	7.5%
FH(+/−) (n=257)	*BRCA1*	BOADICEA	0.704 ± 0.070	0.567 - 0.842	2.908	**0.004**	9.5%
		PennII	0.651 ± 0.070	0.514 - 0.788	2.157	**0.031**	10.0%
	BRCA2	BOADICEA	0.572 ± 0.078	0.420 - 0.725	0.929	0.353	9.8%
		PennII	0.459 ± 0.070	0.322 - 0.597	-0.578	0.563	4.0%
	BRCA1/2	BOADICEA	0.614 ± 0.056	0.503 - 0.725	2.021	**0.043**	9.8%
		PennII	0.558 ± 0.054	0.452 - 0.663	1.069	0.285	8.5%
	BRCA1/2 and *TP53*	BOADICEA	0.614 ± 0.052	0.512 - 0.716	2.193	**0.028**	9.8%
		PennII	0.585 ± 0.051	0.485 - 0.685	1.668	0.095	8.5%

FH(+) = patients with familial history of cancer; FH(−) = patients with no familial history of cancer; FH(+/−) = patients with or without family history (all patients). Relatives with cancer refers to the number of relatives with cancer in the family. Relatives with HBOC core cancer refers to the number of relatives with breast, ovarian, pancreatic, and prostate cancer in the family. The corresponding area under the ROC curve (AUC), standard errors (SE), 95% confidence intervals (CI), z-values, *p*-values, and the optimal cutoff point estimated through the Youden index are presented. All *p*-values <0.05 are in bold.

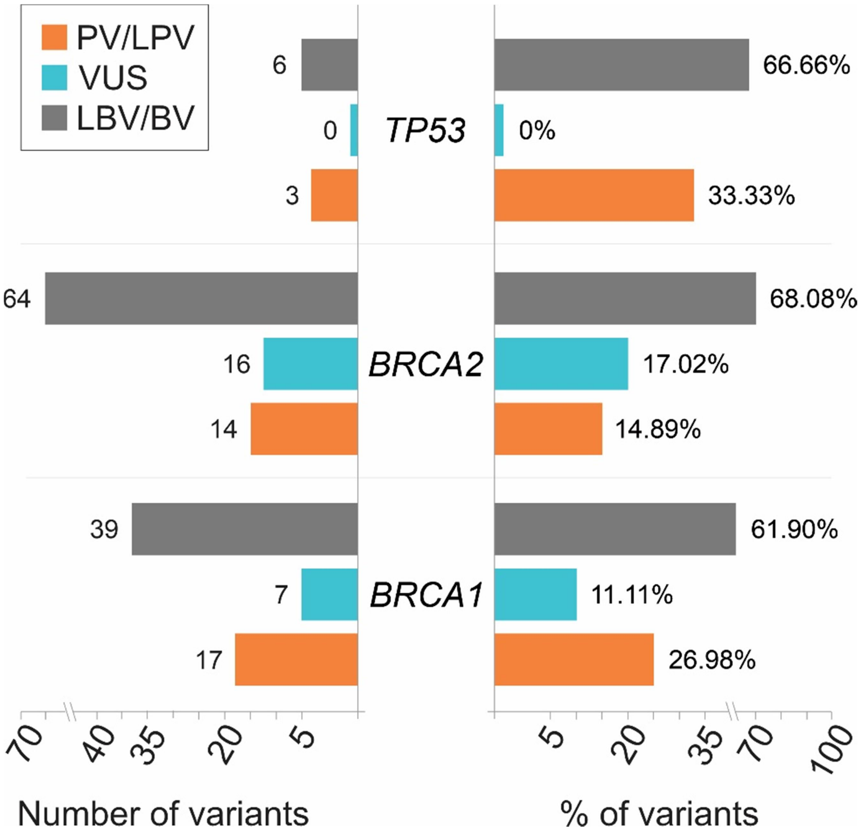

Supplementary Figure S1. Overall distribution and frequency of unique germline variants identified in *BRCA1*, *BRCA2* and *TP53* genes in 257 unrelated patients. PV/LPV: pathogenic or likely pathogenic variants; VUS: variants of uncertain significance; LBV/BV: likely benign or benign variants.

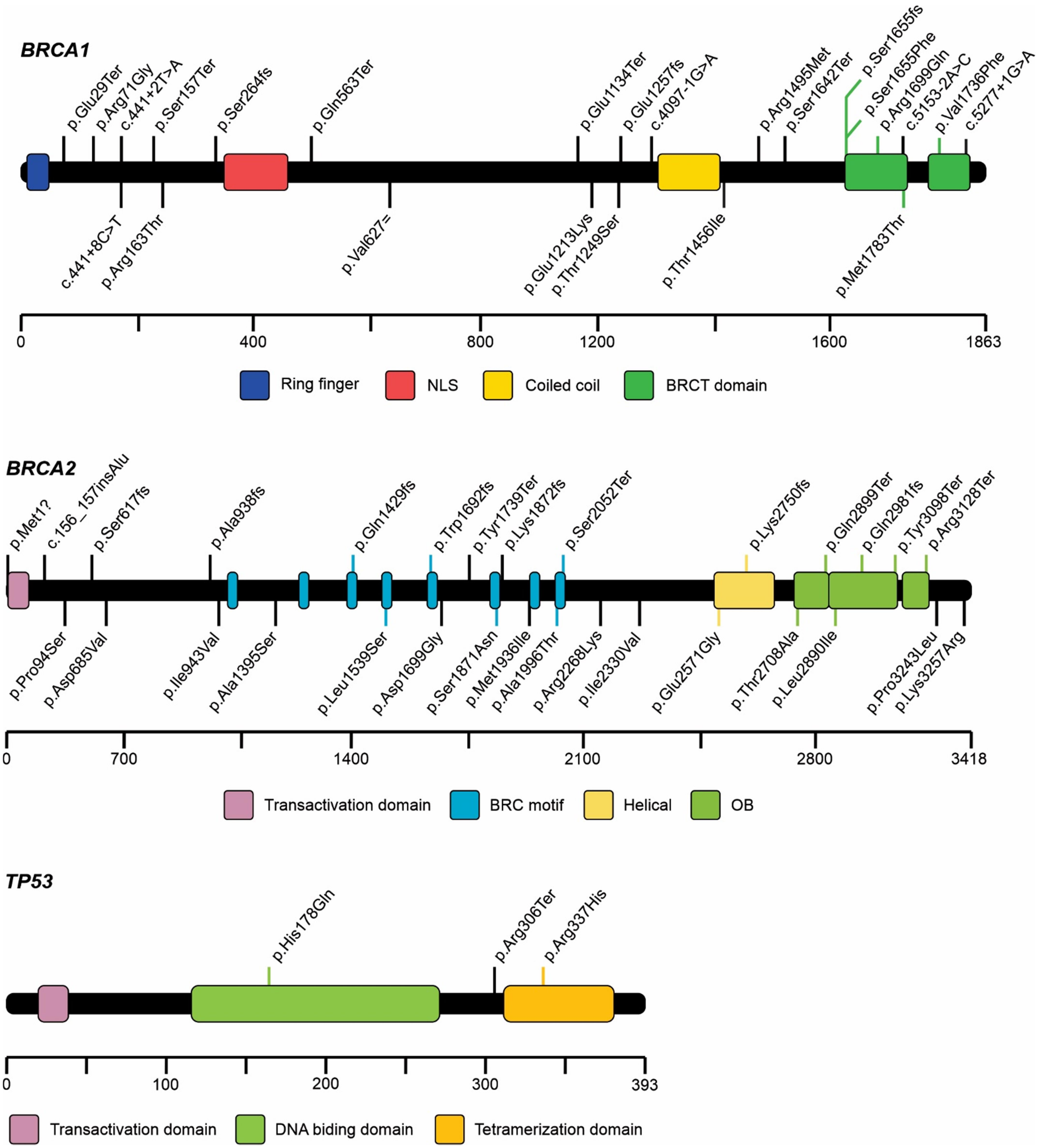

Supplementary Figure S2. Schematic diagram of *BRCA1*, *BRCA2* and *TP53* genes showing the distribution of deleterious variants and variants of uncertain significance. Pathogenic or likely pathogenic variants (PV/LPV) are shown above each gene scheme, while variants of uncertain significance (VUS) are shown below each gene scheme.

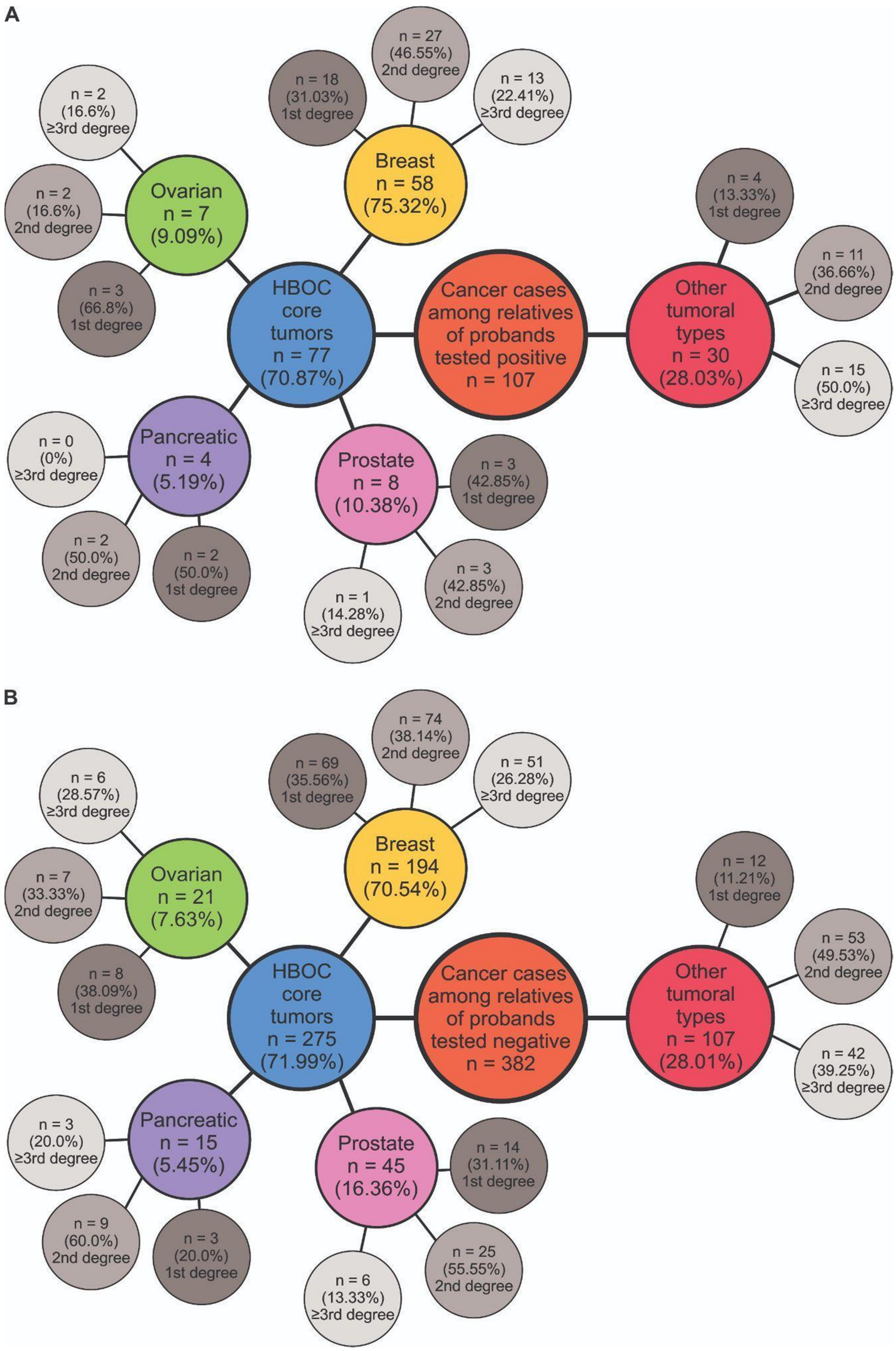

Supplementary Figure S3. Spectrum of main cancer types identified among relatives of the 167 index patients with FH(+): (**A**) relatives of the 30 FH(+) PV/LPV carriers in *BRCA1*, *BRCA2* or *TP53* genes, and (**B**) relatives of the 137 FH(+) non-carriers of PV/LPV in these genes; for details regarding the index patients, see **Table 1**. For convenience, HBOC core tumors refer to breast, ovarian, pancreatic, and prostate cancer.

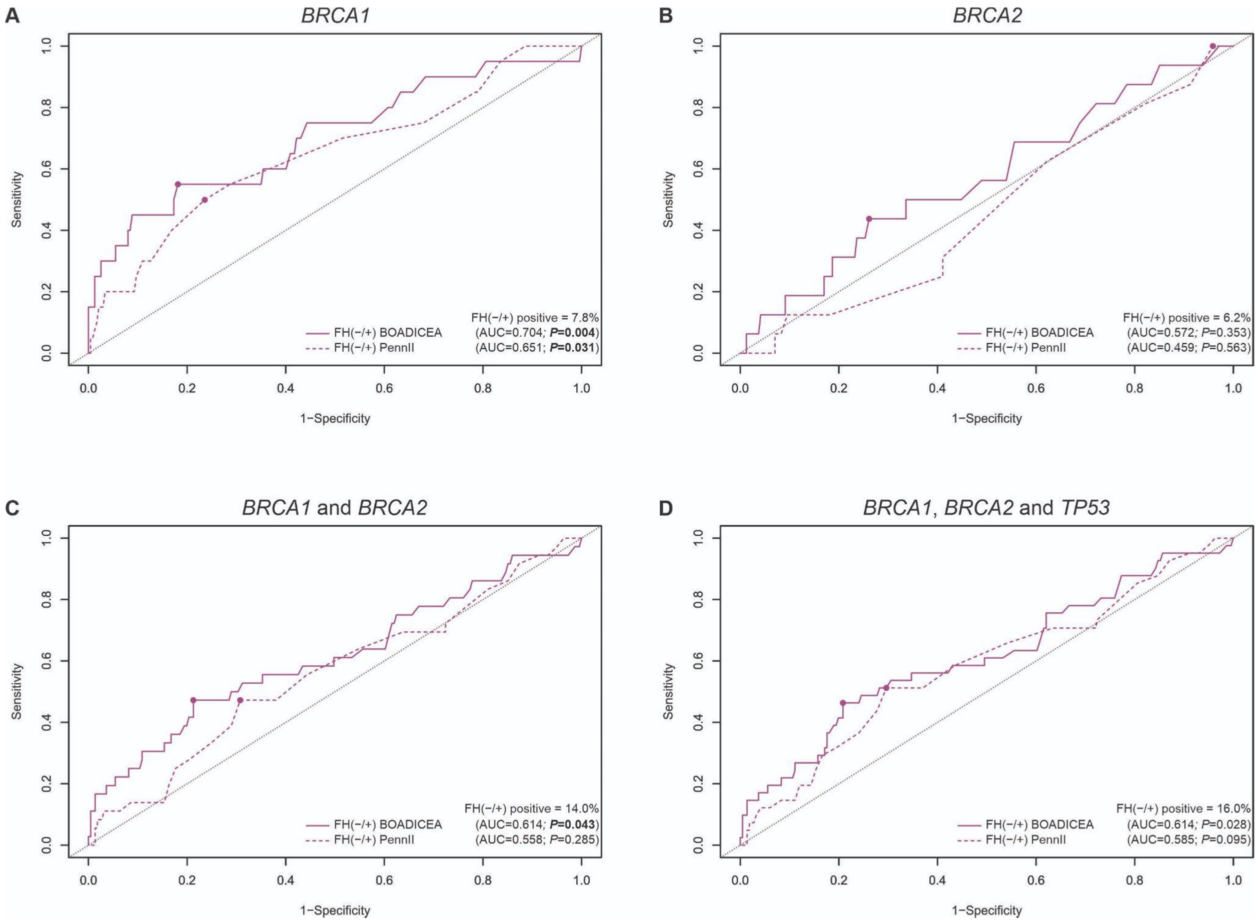

Supplementary Figure S4. Predictive power of detecting PV/LPV carriers (performance) in (A) *BRCA1* only, (B) *BRCA2* only, (C) *BRCA1* and *BRCA2*, or (D) *BRCA1*, *BRCA2* and *TP53*, considering all patients in the cohort: FH(−/+) = patients with or without family history (n=257). Performance was estimated through Receiver Operating Characteristic (ROC) curves for BOADICEA and PennII risk prediction models. FH(−/+) positive refers to the percentage of PV/LPV cases in each case. The corresponding area under the ROC curve (AUC) and *p*-value are presented, as well as the optimal cutoff point (marked by a dot) estimated through the Youden index.
